# Multicenter Analysis of Long-Term Oncologic Impact of Anastomotic Leakage After Laparoscopic Total Mesorectal Excision: The Korean Laparoscopic Colorectal Surgery Study Group

**DOI:** 10.1097/MD.0000000000001202

**Published:** 2015-07-24

**Authors:** Jeonghyun Kang, Gyu-Seog Choi, Jae Hwan Oh, Nam Kyu Kim, Jun Seok Park, Min Jung Kim, Kang Young Lee, Seung Hyuk Baik

**Affiliations:** From the Division of Colorectal Surgery, Department of Surgery, Gangnam Severance Hospital, Yonsei University College of Medicine, Seoul, Korea (JK, SHB); Colorectal Cancer Center, Kyungpook National University Medical Center, School of Medicine, Kyungpook National University, Daegu, Korea (G-SC, JSP); Center for Colorectal Cancer, Research Institute and Hospital, National Cancer Center, Goyang, Gyeonggi-do, Korea (JHO, MJK); and Division of Colorectal Surgery, Department of Surgery, Severance Hospital, Yonsei University College of Medicine, Seoul, Korea (NKK, KYL).

## Abstract

This study aims to validate the oncologic outcomes of anastomotic leakage (AL) after laparoscopic total mesorectal excision (TME) in a large multicenter cohort.

The impact of AL after laparoscopic TME for rectal cancer surgery has not yet been clearly described.

This was a multicenter retrospective study of 1083 patients who underwent laparoscopic TME for nonmetastatic rectal cancer (stage 0–III). AL was defined as an anastomotic complication within 30 days of surgery irrespective of requiring a reoperation or interventional radiology. Estimated local recurrence (LR), disease-free survival (DFS), and overall survival (OS) were compared between the leakage group and the no leakage group using the log-rank method. Multivariate Cox-regression analysis was used to adjust confounding for survival.

The incidence of AL was 6.4%. Mortality within 30 days of surgery occurred in 1 patient (1.4%) in the leakage group and 2 patients (0.2%) in the no leakage group. The leakage group showed a higher LR rate (6.4% vs 1.8%, *P* = 0.011). Five-year DFS and OS were significantly lower in the leakage group than the no leakage group (DFS 71.7% vs 82.1%, *P* = 0.016, OS 81.8% vs 93.5%, *P* = 0.007). Multivariate analysis showed that AL was an independent poor prognostic factor for DFS and OS (hazard ratio [HR] = 1.6; 95% confidence intervals [CI]: 1.0–2.6; *P* = 0.042, HR = 2.1; 95% CI: 1.0–4.2; *P* = 0.028, respectively).

AL after laparoscopic TME was significantly associated with an increased rate of LR, systemic recurrence and poor OS.

## INTRODUCTION

Anastomotic leakage (AL) is a major cause of postoperative mortality and morbidity after rectal cancer surgery, and the incidence of AL ranged from 2.5% to 20%.^1–11^ After the introduction of total mesorectal excision (TME) and spread of this technique to a standard procedure for the management of rectal cancer, it is debatable whether TME in itself results in higher rates of AL. With an increasing proportion of sphincter preserving procedures,^12^ more patients are exposed to the risk of this serious complication.

There are inconsistent data of the effect of AL on oncologic outcomes after rectal cancer surgery. Some authors have demonstrated that AL is associated with an increased rate of local recurrence (LR) or a reduced rate of overall survival (OS).^2–5,13^ In contrast, other investigators reported that the rate of local or distant recurrence remained unaffected in patients with AL.^7–10^ Notably, most of these studies on the impact of AL after rectal cancer surgery were based on patients who underwent open surgery.

Laparoscopic surgery has been increasingly performed for the treatment of rectal cancer. Although recent randomized trials demonstrated short-term benefits or mid-term oncologic safety of laparoscopic rectal cancer surgery, the AL rate between open and laparoscopic surgeries was not changed.^14–16^ It was known that the laparoscopic surgery group showed less immunosuppressed following surgery,^17,18^ so the influence of AL on long-term outcomes might be different between open and laparoscopic surgeries. However, to our knowledge, the oncologic impact of AL for patients who underwent laparoscopic TME has never been investigated on a large-scale study.

Recently, the Korean Laparoscopic Colorectal Surgery Study Group collected clinical data from various hospitals and reported that a low tumor height, male sex, advanced stage, multiple linear stapler firings, preoperative chemoradiotherapy, and perioperative bleeding were independently associated with AL after laparoscopic sphincter-saving TME for rectal cancer.^19^ The results of previous study demonstrated that technical difficulties are still associated with AL in laparoscopic TME for rectal cancer. However, the long-term oncologic impact of AL was not evaluated.

To investigate this issue, we collected another set of patients who underwent laparoscopic TME. Thus, the aim of this study was to evaluate the impact of AL on long-term oncologic outcomes after laparoscopic TME for rectal cancer.

## MATERIALS AND METHODS

### Study Population

As similar to a previous multicenter study, The Korean Laparoscopic Colorectal Surgery Study Group asked individual hospitals to participate in this study. Consecutive patients who performed laparoscopic proctectomy for rectal cancer (pathologic stage 0–III) from January 2006 to December 2009 were collected from 4 hospitals. A total of 1083 patients who had undergone laparoscopic TME and anastomosis for rectal cancer (within 15 cm of the anal verge) were enrolled and composed of the data set of this analysis.

All patients’ preoperative, operative, and postoperative data including survival outcomes were collected using a common menu driven excel file that incorporated precise coding instructions. Perioperative outcomes included age, sex, body mass index (BMI), American Society of Anesthesiologists (ASA) grade, receipt of preoperative chemoradiotherapy, preoperative serum albumin and hemoglobin levels, operative time, perioperative transfusion information, tumor height, type of anastomosis, diverting stoma formation, histopathology details (pT stage, pN stage, pM stage, tumor diameter, circumferential resection margin [CRM], and distal resection margin), hospital stay, morbidity, postoperative chemotherapy and radiotherapy, recurrence site, recurrence date, and survival and deceased date. The sixth edition of the tumor node metastasis (TNM) classification was used to determine the TNM stage in this study. This study was approved by our Institutional Review Board (3-2015-0121).

### Clinical Staging and Preoperative Chemoradiotherapy

Patients were staged with either a combination of appropriate imaging studies such as chest radiography, transrectal ultrasonography, pelvic magnetic resonance imaging (MRI), abdominopelvic computed tomography (CT), chest CT, or 18-FDG positron emission tomography (PET) before the operation.

In general, preoperative chemoradiotherapy was recommended to patients with locally advanced rectal cancer who had a clinical stage T3 or T4 and/or Node (+). However, the decision for preoperative chemoradiotherapy was left to each institution's discretion. Radiotherapy consisted of a total dose of 45 Gy delivered to the pelvis in 25 fractions, followed by a 5.4 to 9 Gy boost to the primary tumor in 3 to 5 fractions given over 5 weeks. The clinical target volume was demarcated as follows: the superior border of the whole pelvis was placed at the lumbosacral junction, the inferior border was placed at the inferior margin of the obturator foramen or 3 cm below the lower margin of the gross tumor, the lateral field border extended 1.5 cm outside the bony pelvis, the anterior border of lateral fields was 3 cm anterior to the gross tumor and shaped to include the internal iliac lymph nodes, and the posterior border of lateral fields extended to encompass all the sacral vertebra. The area that received the prescription dose was specified at the isocenter of the gross tumor volume; the 3-field treatment plan comprised of a 6-MV photon posterior–anterior field, and 6- or 10-MV photon opposed lateral fields with wedges of 45°. 5-Fluorouracil (5-FU) 400 mg/m^2^/day and leucovorin (LV) 20 mg/m^2^/day for 5 days on days 1 to 5 and days 29 to 33 during radiotherapy were delivered with continuous infusion as preoperative chemotherapy regimens. Surgery was performed 6 to 10 weeks after the completion of preoperative chemoradiotherapy. In general, postoperative adjuvant chemotherapy was then added for up to 4 cycles of intravenous 5-FU and LV.

### Surgery and Morbidity

Patients received a mechanical bowel preparation with polyethylene glycol solution the day before surgery. Prophylactic antibiotics were administered just before making the skin incision. Although postoperative care varied slightly across institutions, most surgeons had established a similar protocol for postoperative management and surveillance. Technical procedures for laparoscopic TME, anastomosis technique, creation of a diverting stoma, and drain insertion were already described in a previous study and these procedures were constant in this study.^19^

Postoperative complications were defined as adverse events that occurred within 30 days after surgery. The definition of AL included the discharge of pus or bowel contents through the indwelling drain, pelvic abscess, and local or generalized peritonitis. These criteria included all patients who developed any clinical sign of dehiscence of the anastomosis, regardless of whether a reoperation or any other intervention was required. The diagnosis of AL was usually confirmed by clinical findings, contrast radiography (X-ray or CT scan), or laparotomy.

### Postoperative Adjuvant Chemoradiotherapy

After recovery from surgery, postoperative adjuvant chemoradiotherapy was recommended to patients diagnosed as stage II or III rectal cancer who did not undergo preoperative chemoradiotherapy. For the postoperative adjuvant chemoradiotherapy, adjuvant chemotherapy started within 4 to 8 weeks after curative resection. 5-FU 400 mg/m^2^/day and LV 20 mg/m^2^/day for 5 days were delivered with continuous infusion for 6 cycles with 4-week intervals. Radiotherapy was started at the 3rd cycle of chemotherapy for 5 cycles, and the total amount of the radiation dose was 50.4 to 54 Gy. The radiation technique and boundary were the same as the preoperative chemoradiotherapy. In cases of stage III, other regimens such as FOLFOX or FOLFIRI regimen were used without postoperative radiotherapy according to the policy of each institution.

### Follow-Up

All of the patients had postoperative follow-up visits every 2 to 3 months for 3 years. After 3 years, follow-up visits were reduced to every 6 months until 5 years, and annually thereafter. A physical examination, serum carcinoembryonic antigen level measurement, chest radiography, bone scintigraphies, abdominopelvic CT scans, chest CT, pelvic MRI, or 18-FDG PET scan was performed as indicated according to each institution's policy. All patients undergoing surgery were received close follow-up. Patient follow-up lasted until the cut-off date (June 2014), or when the patient died. The median follow-up period for all the patients was 54 months (range, 1.0–93.0 months).

### Definition of Recurrence

LR was defined as any recurrent tumor growth within the pelvic cavity or perineal area confirmed by clinical, radiological, or pathological evidence. In patients with a suspected LR, pelvic MRI was often performed. For histological confirmation, an imaging-guided biopsy was performed, when possible. However, biopsy confirmation was possible in all but a small number of cases. Overall LR rates are given as the sum of isolated LR and LR with concomitant systemic metastases. Systemic recurrence is defined as recurrence outside the pelvis.

### Statistical Analysis

All statistical analyses were performed using SPSS software, version 20.0 (SPSS, Chicago, IL). Categorical variables were analyzed using the Chi-squared test or Fisher's exact test, and continuous variables were analyzed using the Student *t* test. OS was defined from the date of surgery to the date of death or last follow-up. Disease-free survival (DFS) was defined from the date of surgery to the date of detection of recurrence, last follow-up or death. Differences in survival and recurrences between groups were compared using the Kaplan–Meier method and tested with the log-rank test. For cumulative LR calculations, duration of follow-up was calculated from the day of surgery and analyzed by the reverse Kaplan–Meier method.^20^ All variables *P* < 0.05 on univariate analysis were initially entered into the multivariate analysis. Factors associated with DFS and OS were analyzed by a Cox-proportional hazards regression analysis done by a forward stepwise selection of variables. *P* < 0.05 was considered statistically significant.

## RESULTS

Among 1083 enrolled patients, AL occurred in 69 patients (6.4%). The AL of the patients was categorized into the leakage group, and the others were categorized into the no leakage group.

### Clinicopathological Characteristics

Clinical characteristics of the patients in the 2 groups are listed in Table 1. No significant difference was observed in age, BMI, ASA grade, preoperative hemoglobin and albumin level, and rate of preoperative chemoradiotherapy. However, the leakage group showed male predominance and lower tumor height compared with the no leakage group (75.4% vs 61.3%, *P* = 0.020, 7.8 cm vs 9.3 cm, *P* < 0.001, respectively).

**TABLE 1:**
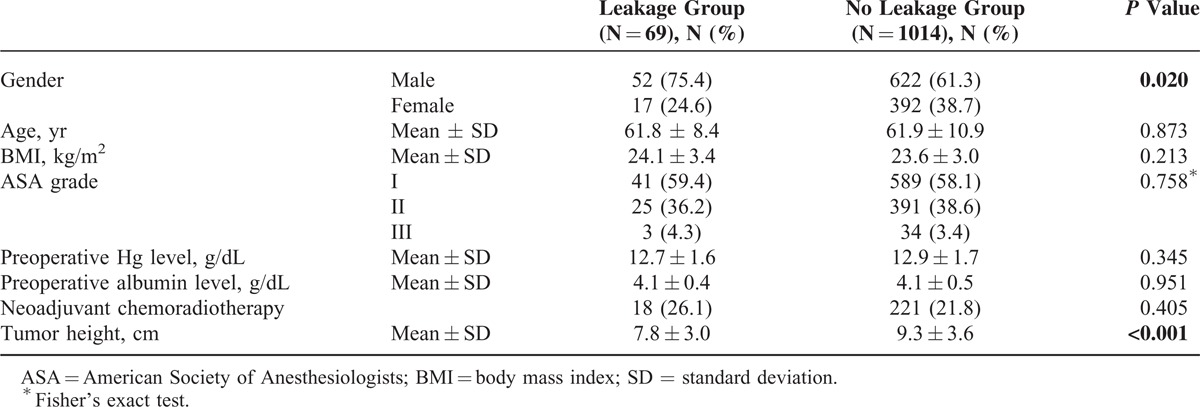
Patient Characteristics

### Perioperative Outcomes

Perioperative outcomes of surgery are listed in Table 2. Operation type was not different between the 2 groups. Mean operation time was significantly longer in the leakage group compared with the no leakage group (*P* = 0.006). Rate of performing fecal diversion was similar between the 2 groups. Blood transfusion during the operation or the day of operation was more commonly performed in the leakage group (10.1% vs 2.9%, *P* = 0.006). Length of hospital stay was significantly longer in the leakage group compared with the no leakage group (30.1 days vs 9.9 days, *P* < 0.001).

**TABLE 2:**
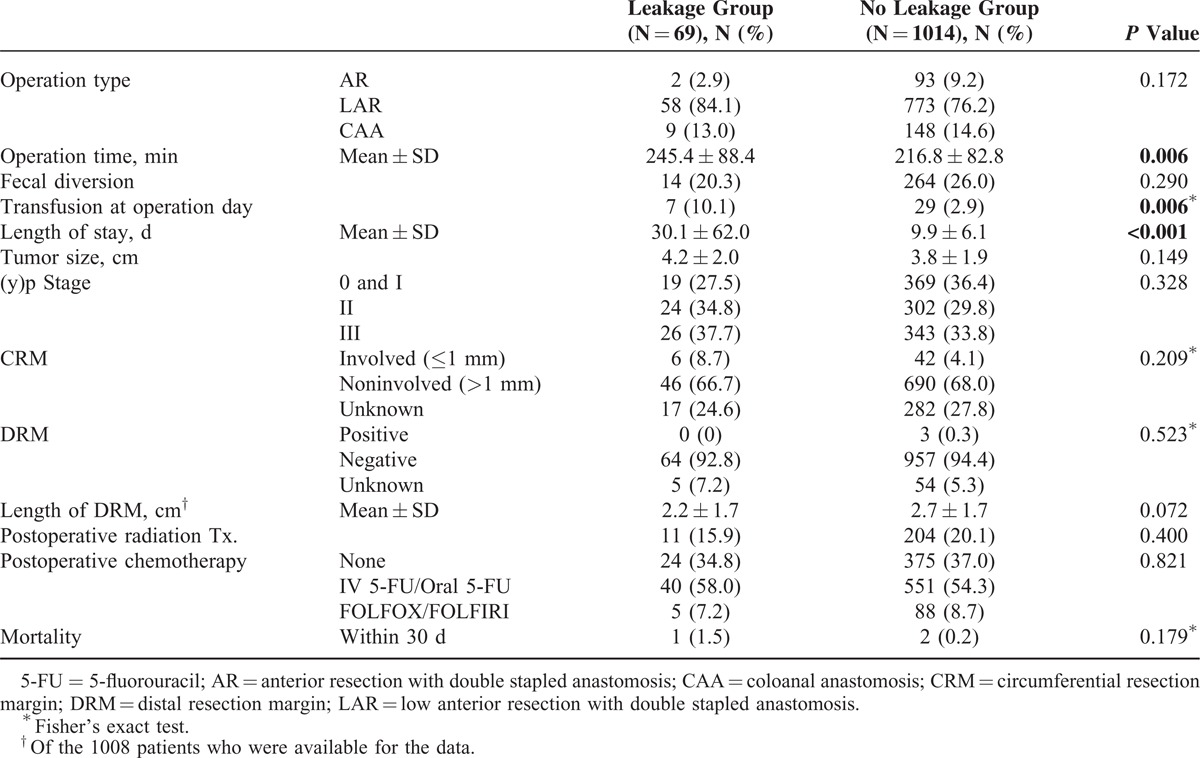
Perioperative Clinicopathological Outcomes

No significant difference was observed in tumor size, (y)p Stage, distal resection margin, and CRM involvement rate. However, CRM status was available in 784 patients (72.4%). The rate of performing postoperative radiotherapy was similar between the 2 groups. There was no difference of chemotherapy regimen between the 2 groups. There was 3 postoperative mortalities (1 in the leakage group and 2 in the no leakage group, *P* = 0.179)

### Oncologic Outcomes

**FIGURE 1:**
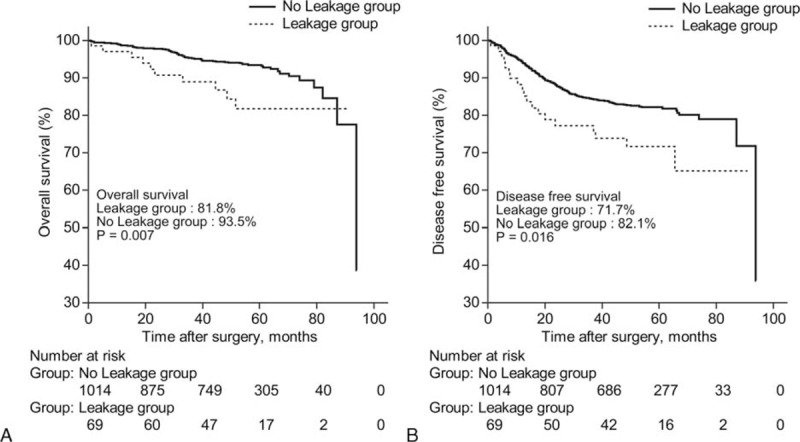
Five-year overall survival (OS) and disease-free survival (DFS) between the leakage group and the no leakage group. The leakage group showed poor 5-year OS (A) and DFS (B) than the no leakage group (*P* < 0.05, for all).

**FIGURE 2:**
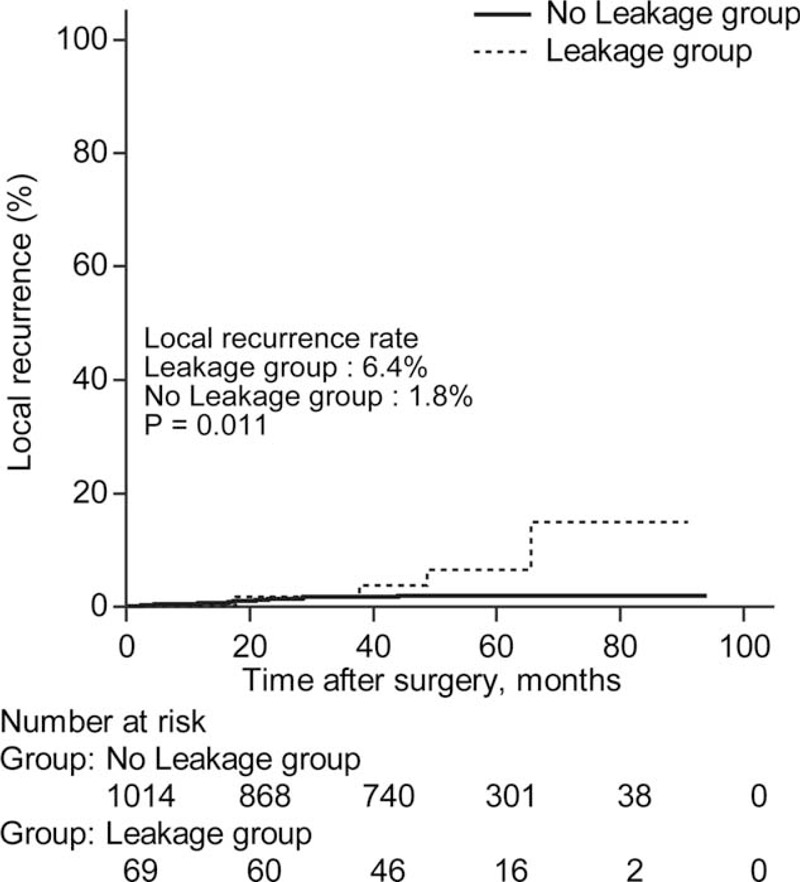
Cumulative 5-year local recurrence rate between the leakage group and the no leakage group. Cumulative 5-year local recurrence rate was significantly higher in the leakage group than the no leakage group (the leakage group vs the no leakage group; 6.4% vs 1.8%, *P* = 0.011).

The 5-year DFS and OS was significantly lower in the leakage group than the no leakage group (DFS 71.7% vs 82.1%, *P* = 0.016, OS 81.8% vs 93.5%, *P* = 0.007) (Figure 1). The overall 5-year cumulative LR rate was 6.4% in the leakage group and 1.8% in the no leakage group (*P* = 0.011) (Figure 2).

For DFS, age, preoperative hemoglobin level, preoperative chemoradiotherapy, tumor height, perioperative transfusion, tumor size, stage, CRM, postoperative chemotherapy, and AL were proved to be significant prognostic factors in univariate analysis (Table 3). Among them, age, preoperative chemoradiotherapy, stage, and AL remained as prognostic factors in multivariate analysis. For OS, age, ASA grade, preoperative hemoglobin level, stage, CRM, and AL were proved to be significant prognostic factors in univariate analysis (Table 3). Among them, age, ASA grade, stage, CRM, and AL remained as significant factors associated with OS in multivariate analysis. In brief, multivariate analysis showed that AL was an independent poor prognostic factor for DFS and OS (hazard ratio [HR] = 1.6; 95% confidence intervals [CI]: 1.0–2.6; *P* = 0.042, HR = 2.1; 95% CI: 1.0–4.2; *P* = 0.028, respectively) (Table 4).

**TABLE 3:**
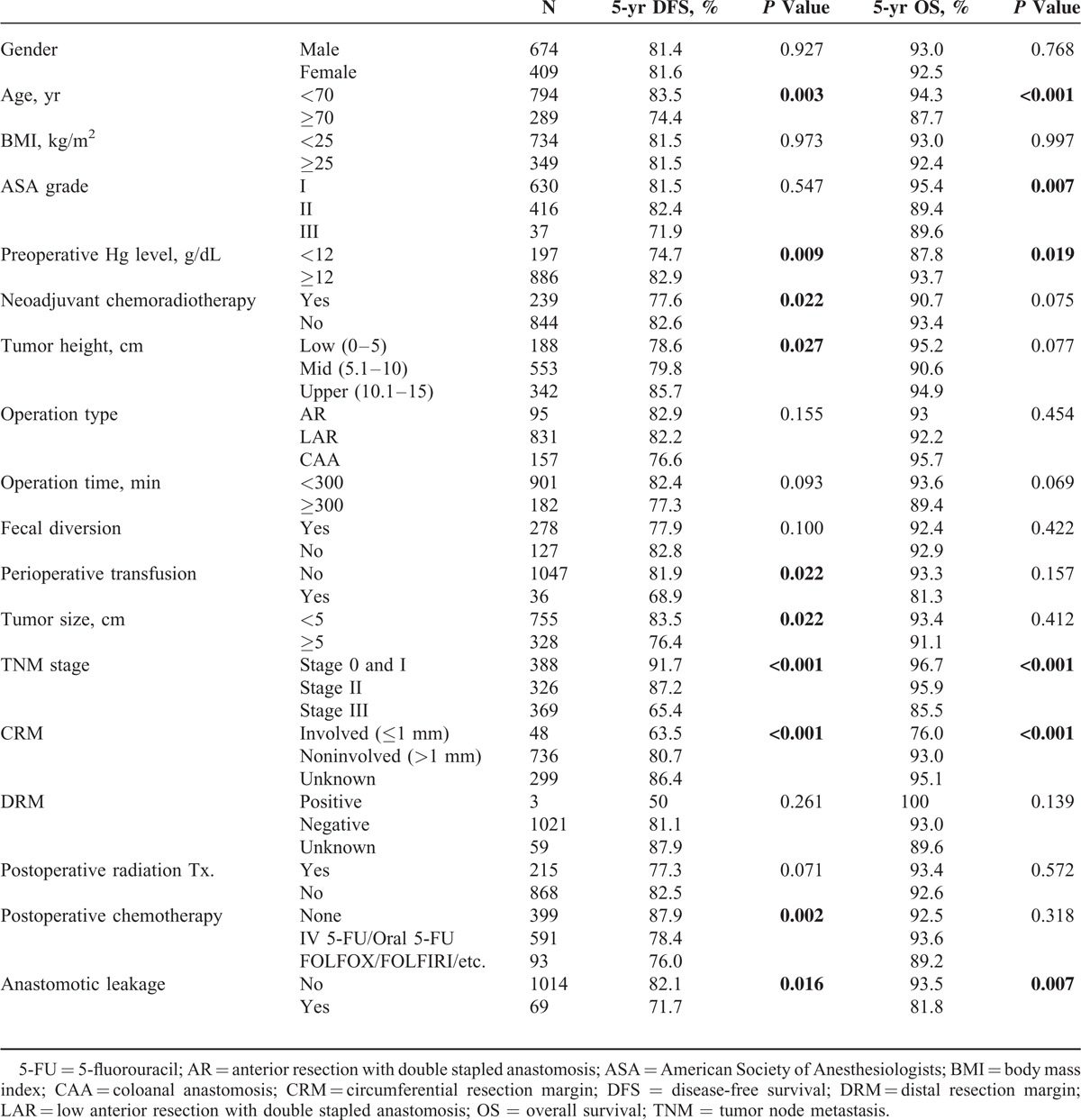
Univariate Analysis of Prognostic Factors

**TABLE 4:**
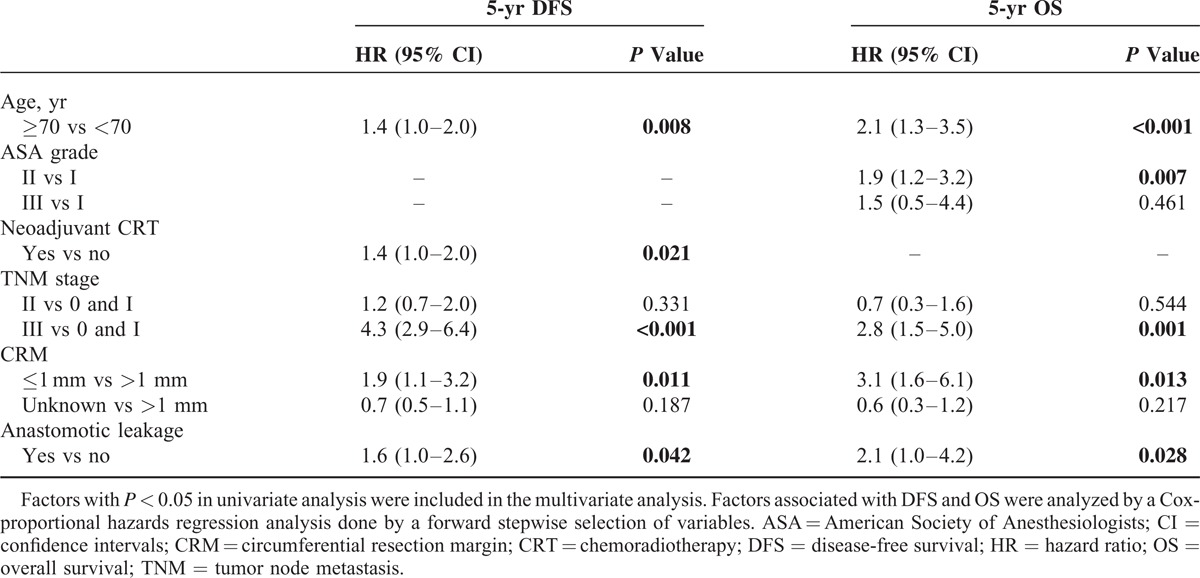
Multivariate Analysis of Prognostic Factors

## DISCUSSION

This multicenter analysis demonstrated that AL after laparoscopic TME with sphincter preservation in patients with rectal cancer resulted in inferior oncologic outcomes with respect to DFS and OS.

Laparoscopic surgery in management of rectal cancer could provide an enhanced benefit to patients in terms of postoperative early recovery and reduced pain.^21^ Long-term oncologic outcome of laparoscopic rectal cancer surgery is recently reported to be comparable to that of open surgery.^14,22^ Thus, application of laparoscopy would be increased for rectal cancer surgery. However, in a study comparing open, laparoscopic, and robotic surgeries for mid and low rectal cancers, the AL rate was significantly higher in the laparoscopic surgery group than the other 2 groups.^23^ It is argued that difficulties in perpendicular transection of the rectum and increased number of linear stapler firings in laparoscopic surgery might result in the high incidence of AL.^19,23–25^ Currently, the impact of AL on LR or systemic recurrence after laparoscopic TME has not been evaluated in a large-scale study design. Most previous studies focusing on the oncologic outcomes after AL in rectal cancer included patients who underwent open surgery. As far as we know, the strength of our study is that this study might be one of the first studies focusing on this issue.

The impact of AL on long-term oncologic outcomes for patients with rectal cancer is contradictory. In a large-scale population-based cohort study, AL did not result in an increased LR rate.^8^ In a single center-based study, clinical leakage was not associated with time to LR, DFS, or OS.^7,10^ In contrast, many investigators reported an increased LR rate or reduced OS rate after AL.^2–6,13^ One of the possible explanations for this discrepant oncologic effect of AL after rectal cancer surgery might be the fact that the definition of AL has not been standardized.^8,26^ It is well known that delayed leakage, such as rectovaginal fistula, pelvic abscess, or radiologic detected subclinical leakage showed different clinical manifestations compared with AL occurring in immediate postoperative periods.^27^ Although the International Study Group of Rectal Cancer suggested the 3 grading system of AL in 2010,^28^ most previous studies used their own definition of AL. For example, Ptok et al^3^ divided AL patients into “surgically treated leakage” and “nonsurgically treated leakage” and compared these 2 groups with a “no leakage group.” Smith et al^7^ defined clinical leakage as an anastomotic event requiring intervention or interventional radiology within 60 days of surgery. Such differences could impact the association of AL with recurrence or survival.

In the present study, the leakage group showed worse prognosis in LR, systemic recurrence, and OS. The underlying mechanism of the poor oncologic impact of AL cannot be fully accounted for by the result of our study. Walker et al^6^ speculated that the poor oncologic outcomes of patients with AL may be attributed not only to the higher rate of morbidity but also to some unknown inflammation-related immunologic stimulation of cancer recurrence. Recently, Salvans et al^29^ reported that postoperative peritoneal fluid from infected patients enhanced both cell migration and cell invasion capacities of cancer cell lines, which might be one of the possible mechanisms responsible for the association between postoperative peritoneal infection and tumor recurrence. Another potential reason is that, considering the presence of viable tumor cells in the bowel lumen of patients with rectal cancer,^30,31^ AL may lead to extraluminal implantation of exfoliated cancer cells remaining in the bowel lumen.^3^

Significant changes were introduced in the management of colorectal cancer in terms of surgical technique and adjuvant therapy. The survival rate of colorectal cancer has gradually improved since 1975 to 2006 in the USA.^32^ A single center-based study by Smith et al^7^ demonstrated no difference of oncologic results in patients with an AL compared with those without it. In detail, they included patients treated from 1991 to 2010. Thus, different treatment strategies owing to a long span of enrolled periods are inevitable, which might be act as a bias. In addition, as the authors commented, their study had possibilities of underpowered analysis due to relatively few events of AL and LR. When we analyzed the impact of AL on oncologic outcomes, one of the main limitations was that the small number of AL patients hindered it as a meaningful parameter of multivariate analysis for survival. Our large-scale multicenter analysis enrolled recently treated patients in a short span from 2006 to 2009. The treatment modalities for rectal cancer during the study periods were already well established. Also preoperative chemoradiotherapy, TME, and postoperative adjuvant treatment were included. In addition, this study has strengths in including adequate events of AL to evaluate the primary end point.

Nevertheless, this retrospective study design still has several potential limitations. In this large-scale multicenter evaluation, the AL rate was 6.4%, which was almost similar to the results of a previous study.^19^ As described in our earlier observational study, it is impossible to obtain the actual AL rate including clinical leakage and subclinical leakage, because radiologic studies such as barium enema before closure of a stoma were not routine procedures in our study cohort.^19^ In this study, the diagnosis of AL was dependent on clinical presentations, which might be a potential limitation of this study. Second, important clinicopathological factors such as CRM involvement were not available in whole study populations. CRM involvement is known to be one of the most important prognostic factors in rectal cancer.^33^ Third, the indications of applying laparoscopic surgery, preoperative chemoradiotherapy, or making a diverting stoma during the surgery were left to the discretion of the surgeon. Although this reflects daily clinical practice, it could cause a selection bias. Finally, in this study, the 30-day mortality rate was 1.4% in the leakage group and overall 30-day mortality rate in this cohort was 0.2%. It is known that the mortality rate after AL ranged from 4% to 18%.^3,11,13,34^ This result might reflect a recent development of postoperative care and intensive care unit management. In addition, our enrolled patients were treated in highly qualified centers for laparoscopic surgery in our nation. Thus, it remains unanswered that our results are reproducible in general practice.

In conclusion, AL is associated with increased LR and reduced OS after laparoscopic TME with sphincter preservation. Therefore, surgeons should be more cautious of reducing the AL rate when performing laparoscopic TME.
